# Serum microRNA-1233 is a specific biomarker for diagnosing acute pulmonary embolism

**DOI:** 10.1186/s12967-016-0886-9

**Published:** 2016-05-05

**Authors:** Thorsten Kessler, Jeanette Erdmann, Baiba Vilne, Petra Bruse, Volkhard Kurowski, Patrick Diemert, Heribert Schunkert, Hendrik B. Sager

**Affiliations:** Deutsches Herzzentrum München, Klinik für Herz- und Kreislauferkrankungen, Technische Universität München, Lazarettstr. 36, 80636 Munich, Germany; Institut für Integrative und Experimentelle Genomik, Universität zu Lübeck, Lübeck, Germany; Deutsches Zentrum für Herz-Kreislauf-Forschung (DZHK) e.V., partner site Hamburg/Lübeck/Kiel, Lübeck, Germany; Innere Medizin, DRK-Krankenhaus Mölln-Ratzeburg, Ratzeburg, Germany; Medizinische Klinik II, Westküstenklinikum, Heide, Germany; Deutsches Zentrum für Herz-Kreislauf-Forschung (DZHK) e.V., partner site Munich Heart Alliance (MHA), Munich, Germany

**Keywords:** Pulmonary embolism, Non ST-segment elevation myocardial infarction micro-RNA, Biomarker

## Abstract

**Background:**

Circulating microRNAs (miRNAs) emerge as novel biomarkers in cardiovascular diseases. Diagnosing acute pulmonary embolism (PE) remains challenging due to a diverse clinical presentation and the lack of specific biomarkers. Here we evaluate serum miRNAs as potential biomarkers in acute PE.

**Methods:**

We enrolled 30 patients with acute, CT (computed tomography)-angiographically confirmed central PE and collected serum samples on the day of emergency room admission (1st day) and from 22 of these patients 9 months thereafter. For comparison, we examined serum samples from patients with acute non ST-segment elevation myocardial infarction (NSTEMI, n = 30) and healthy individuals (n = 12).

**Results:**

We randomly selected 16 out of 30 PE patients and screened sera from the acute (1st day) and chronic stages (9 months) for 754 miRNAs using microarrays and found 37 miRNAs to be differentially regulated. Across all miRNAs, miRNA-1233 displayed the highest fold change (FC) from acute to chronic stage (log_2_FC 11.5, p < 0.004). We validated miRNA-1233 by real-time quantitative polymerase chain reaction (RT-qPCR). In acute PE (1st day) we found elevated levels of miRNA-1233 in comparison to NSTEMI (log_2_FC 5.7, p < 0.0001) and healthy controls (log_2_FC 7.7, p < 0.0001). miRNA-1233 differentiated acute PE from NSTEMI patients and healthy individuals with 90 and 90 % sensitivity, and 100 and 92 % specificity [area under the curve (AUC) 0.95, p < 0.001 and 0.91, p < 0.001], respectively.

**Conclusions:**

This is the first report that identifies a miRNA that allows distinguishing acute PE from acute NSTEMI and healthy individuals with high specificity and sensitivity.

## Background

Acute pulmonary embolism (PE) is a common cardiovascular emergency with a high incidence of morbidity and mortality [1, 2]. Together with deep vein thrombosis (DVT), PE is the third most frequent cardiovascular disease in western countries with an annual incidence of 100–300 per 100,000 individuals [3]. PE is difficult to diagnose and frequently missed due to a variable clinical presentation including dyspnea at rest or on exertion, chest pain and syncope [4, 5]. Patients with acute myocardial infarction (MI) also often present chest pain and shortness of breath, consequently acute MI is one of acute PE’s most frequent differential diagnoses. The work-up for diagnosing PE can comprise biomarkers (such as the fibrin degradation product D-dimer), echocardiography, venous compression ultrasonography and radiologic imaging with computed tomography angiography, and ventilation–perfusion scintigraphy [1, 6]. Although widely used in patients with suspected PE, D-dimer tests exhibit a high sensitivity, but fall short of being specific for diagnosing acute PE [7]. Hence, the introduction of novel biomarkers with a superior diagnostic accuracy would strongly facilitate diagnosing acute PE.

MicroRNAs (miRNAs) are small (~22 nucleotides), non-coding, single-stranded RNAs which can inhibit protein synthesis by negatively regulating gene expression via translational repression or mRNA degradation [8–10]. Often, miRNA expression is cell or tissue specific and their expression pattern can be altered upon initiation of pathologic processes. miRNAs can be detected in the circulation (serum or plasma) in a stable form [11] and thus represent attractive biomarkers for cardiovascular diseases [12–22].

In our study, we screened sera of patients with acute PE at different time points during the course of disease for differentially regulated miRNAs. We validated three selected miRNAs by qPCR and then tested their specificity and sensitivity for accurately diagnosing acute PE. This is the first report that identifies a profile of circulating miRNAs that allows distinguishing acute PE from acute non ST-segment elevation myocardial infarction (NSTEMI), acute DVT, chronic non-thromboembolic pulmonary hypertension (PH) and healthy individuals with high specificity and sensitivity.

## Methods

### Patients of the study group

We enrolled patients presenting with acute symptom onset at the emergency department at the University Hospital of Schleswig–Holstein, Campus Lübeck, Germany who were diagnosed with central PE (location of the embolism in the pulmonary trunk and/or main pulmonary artery/ies) by CT-pulmonary angiography. Serum samples were taken at the emergency department on the admission day (1st day), as well as on the third and on the fifth day of the hospital stay. We recalled 22 patients 9 ± 1.5 months after the hospital stay and took a fourth serum sample.

### Patients of the control groups

For comparison, we enrolled patients that presented at the emergency department with acute NSTEMI. Furthermore, we enrolled patients with acute DVT, patients with chronic non-thromboembolic PH and healthy individuals (colleagues in the lab without any current of past medical condition).

### Ethics, consent and permissions

Patients gave informed consent to participate in this study as part of the Lübeck Registry of Structural Heart Disease [23], approved by the ethics committee of the University of Lübeck (No. 04-041).

### Consent to publish

Patients gave informed consent for publishing results of this study as part of the Lübeck Registry of Structural Heart Disease [23].

### RNA isolation

Blood samples were allowed to coagulate at room temperature and then immediately centrifuged at 1500 g for 15 min. The supernatant was transferred to RNase/DNase-free tubes and stored at −80 °C. RNA isolation was performed by using a miRNeasy Mini Kit (Qiagen, Hilden, Germany) with combining phenol/guanidine-based lysis and silicamembrane-based purification of RNA, according to the manufacturer’s instructions. In brief, 300 µl of serum were homogenized in 900 µl of QIAzol lysis reagent (Qiagen, Hilden, Germany). After adding 240 µl chloroform (Merck Millipore, Germany), the homogenate was separated by centrifugation. The upper (aqueous) phase was extracted and 100 % ethanol (Merck Millipore, Germany) was added. Samples were then applied to miRNeasy Mini spin columns. RNA was eventually eluted in RNase-free water. To normalize for the miRNA content, we supplemented samples with 10 nM miRNA 39 from *Caenorhabditis elegans* (cel-miR-39, Applied Biosystems, Foster City, CA, USA) as spiked-in control, after adding the QIAzol, as described previously [24, 25].

### Serum miRNA profiles

We screened sera from PE patients for 754 miRNAs using TaqMan low-density miRNA microarrays (human miRNA A V2 and human miRNA B V3, Applied Biosystems, Foster City, CA, USA). Reverse transcription (RT) and pre-amplification steps were performed using the same volume of total RNA, according to the manufacturer’s protocol. Real-time quantitative polymerase chain reaction (RT-qPCR) was performed using the 7900 HT Fast Real-Time PCR System (Applied Biosystems, Foster City, CA, USA); results were expressed as Cts (cycle threshold, with the baseline set to 0.2).

### Validation of findings using miRNA RT-qPCR

Based on the results from the screening experiments, we selected miRNAs for further RT-qPCR validation. After RNA extraction, and pre-amplification (carried out as described above), individual miRNA expression was determined using TaqMan microRNA Assays (Applied Biosystems, Foster City, CA, USA), according to manufacturer’s instructions. We used the following TaqMan probes: hsa-miR-1233-002768, hsa-miR-27a#-002445 and hsa-miR-134-001186 (Applied Biosystems, Foster City, CA, USA). In individual TaqMan microRNA Assays, cel-miRNA-39 was used for normalization.

### Computational methods

#### miRNA screening

miRNA array data analysis was performed using R/Bioconductor packages [26]. Raw threshold cycle (Ct) values were first processed by replacing ‘Undetermined’ Ct values with Ct = 50.0 and thereafter filtered in a way that we excluded miRNAs where the median Ct was ≤35.0 in at least one of the comparison groups (‘acute’ vs. ‘chronic’). After filtering, raw Ct values were converted into relative quantities (RQ) by using a formula RQ = E^ΔCt^, where PCR efficiency (E) was assumed to be 100 % [27], reflected by a value of two for the base E of the exponential function. Median normalization was used in order to obtain normalized relative quantities (NRQ) by dividing each RQ with the overall median of the corresponding sample (‘acute’ vs. ‘chronic’). For two-way comparisons, the limma [28] t-statistic approach with Benjamini–Hochberg (BH; FDR) multiple testing correction [29] was used to select the differentially expressed miRNAs from the median-normalized data. miRNAs were defined as differentially expressed if they had a −1 ≥ log_2_FC ≥ 1 and *P* value ≤0.15 across the comparison groups (‘acute’ vs. ‘chronic’).

#### miRNA validation

Statistical analyses were carried out using GraphPad Prism software, version 6 (GraphPad Software, Inc.). Results are displayed as mean ± standard error of mean (S.E.M.), if not noted otherwise. First, values were tested for outliers (ROUT method, Q = 1 %) and for Gaussian distribution (D’Agostino-Pearson omnibus normality test). For comparing more than two groups, an ordinary one-as applied to parametric data. For non-parametric data, a Kruskal–Wallis test was performed, followed by a Dunn’s test for multiple comparisons. P values of <0.05 indicated statistical sway ANOVA test, followed by a Sidak’s test for multiple comparisons, significance.

#### ROC curve analyses

All analyses were performed using R [30]. The diagnostic accuracy of individual miRNAs and their combinations was assessed using logistic regression and multinomial logistic regression analysis, respectively. The trade-offs between the specificity and sensitivity measures were then assessed using the receiver operating characteristic (ROC) curves (pROC package [31]). The area under the ROC curve (AUC) was used as summary measure to average the detection accuracy across the spectrum of test values. The AUC for each prediction was compared to random classifier (AUC = 0.5) using the Wilcoxon signed rank test, as implemented in MKmisc http://crantastic.org/packages/MKmisc. The maximum value of the Youden’s index (J = Sensitivity + Specificity − 1) for each ROC curve was used for selecting the optimal cut-off point for the diagnostic tests. The positive likelihood ratio (LR+) was calculated as sensitivity/(1 − specificity), the negative likelihood ratio (LR−) as (1 − Sensitivity)/Specificity.

## Results

### Patients and serum sampling

**Fig. 1:**
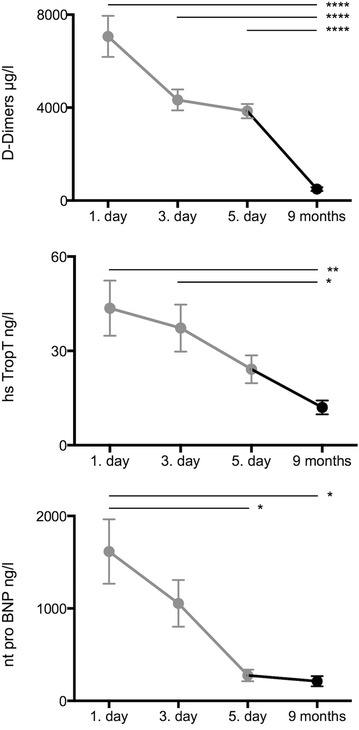
Biomarker time course. Levels of the biomarkers D-Dimers, high sensitive (hs) TroponinT and n-terminal (nt) pro brain natriuretic peptide (BNP) during the course of pulmonary embolism (PE, 1st day: presentation at the emergency department, 3rd and 5th day during the hospital stay (n = 30) and 9 ± 1.5 months thereafter (n = 22), mean ± SEM, *p < 0.05, **p < 0.01, ****p < 0.0001)

We enrolled 30 patients (age 62 ± 14, mean ± SD, 57 % male, all Caucasians) who presented with acute symptom onset at the emergency department and were here diagnosed with central PE (location of the embolism in the pulmonary trunk and/or main pulmonary artery/ies) by computed tomography (CT)-pulmonary angiography (Table 1). The majority of patients presented with shortness of breath (81 %) and/or chest pain (33 %). In most patients the cause of PE remained idiopathic. The time course of biomarkers D-dimers, high sensitive troponin T and n-terminal (nt) pro brain natriuretic peptide (BNP) is displayed in Fig. 1. Serum samples were taken at the emergency department on the admission day (1st day), as well as on the third and fifth day of the hospital stay. We re-called 22 (50 % male patients) of these 30 patients 9 ± 1.5 months (mean ± SD) after the hospital stay and collected a fourth serum sample (9 months). For comparison, we enrolled 30 age- and gender-matched patients that presented at the emergency department with acute non ST-segment elevation myocardial infarction (NSTEMI, mean age ± SD: 64 ± 13, 57 % males, Table 2). Furthermore, we enrolled six age-matched patients with acute DVT (mean age ± SD: 66 ± 10, 67 % males) confirmed by compression ultrasonography without any clinical or apparative signs of concomitant PE (no dyspnea, no chest pain, normal ECG, normal right heart echocardiography, normal blood gas analysis), 15 age-matched patients with chronic non-thromboembolic pulmonary hypertension (PH, mean age ± SD: 65 ± 13, 47 % males), and 12 healthy individuals (mean age ± SD: 31 ± 6, 50 % males).

**Table 1:** Patients’ characteristics

n	30
Age (mean ± SD)	62 ± 14
Male	17/30 (57 %)
CTA confirmed central PE (pulmonary trunk, main pulmonary arteries)	100 %
Risk stratefication (PE-related early mortality rate)	
High (>15 %)	4/30 (13 %)
Intermediate (3–15 %)	15/30 (50 %)
Low (<1 %)	11/30 (37 %)
Symptoms	
Dyspnoea	24/30 (81 %)
Chest pain	10/30 (33 %)
Cough	3/30 (10 %)
Syncope	3/30 (10 %)
Haemoptysis	2/30 (7 %)
Collaps with subsequent CPR	4/30 (13 %)
Idiopathic DVT/PE	13/30 (43 %)
Secondary DVT/PE	
Malignancy	4/30 (13 %)
Post surgery	4/30 (13 %)
Immobilisation	2/30 (7 %)
Thrombophilia	1/30 (3 %)
Previous DVT/PE	2/30 (7 %)
Oral contraceptive therapy	2/30 (7 %)
Obesity (BMI ≥ 30 kg/m^2^)	12/30 (40 %)
US confirmed DVT	25/30 (83 %)

Characteristics of acute pulmonary embolism (PE) patients at the time of the emergency room presentation (1st day)

*CTA* computed tomography (pulmonary) angiography, *CPR* cardiopulmonary resuscitation, *DVT* deep vein thrombosis, *BMI* body mass index, *US* ultrasound, *SD* standard deviation

**Table 2:** Patients’ characteristics

	Acute PE	Acute NSTEMI	p
Age (y)	62 ± 14	64 ± 13	0.57
Male gender (%)	57	57	1.00
Onset to sampling < 24 h (%)	63	70	0.58
BMI (kg/m^2^)	30.6 ± 7.7	27.9 ± 9.3	0.23
LV-EF (%)	60 ± 2	57 ± 7	0.03
*CV risk factors*			
Hypertension (%)	63	81	0.15
Hyperlipidemia (%)	17	41	0.04
Smoking (%)	10	52	<0.001
Diabetes mellitus (%)	20	22	0.75
Positive family history (%)	3	33	<0.01
*CV history*			
Non-obstructive CAD (%)	17	3	0.09
Obstructive CAD (%)	0	6	0.15
Previous PCI/CABG (%)	0	6	0.15
Previous MI (%)	0	6	0.15
Resuscitation (%)	0	0	1.00
Atrial fibrillation (%)	0	3	0.31
Previous stroke (%)	3	3	1.00

Patients’ characteristics of the acute pulmonary embolism (PE) and acute non ST-segment elevation myocardial infarction (NSTEMI) groups

*BMI* body mass index, *LV*-*EF* left ventricular ejection fraction, *CV* cardiovascular, *CAD* coronary artery disease, *PCI* percutaneous coronary intervention, *CABG* coronary artery bypass grafting, *MI* myocardial infarction

### Serum miRNA screening and validation

**Fig. 2:**
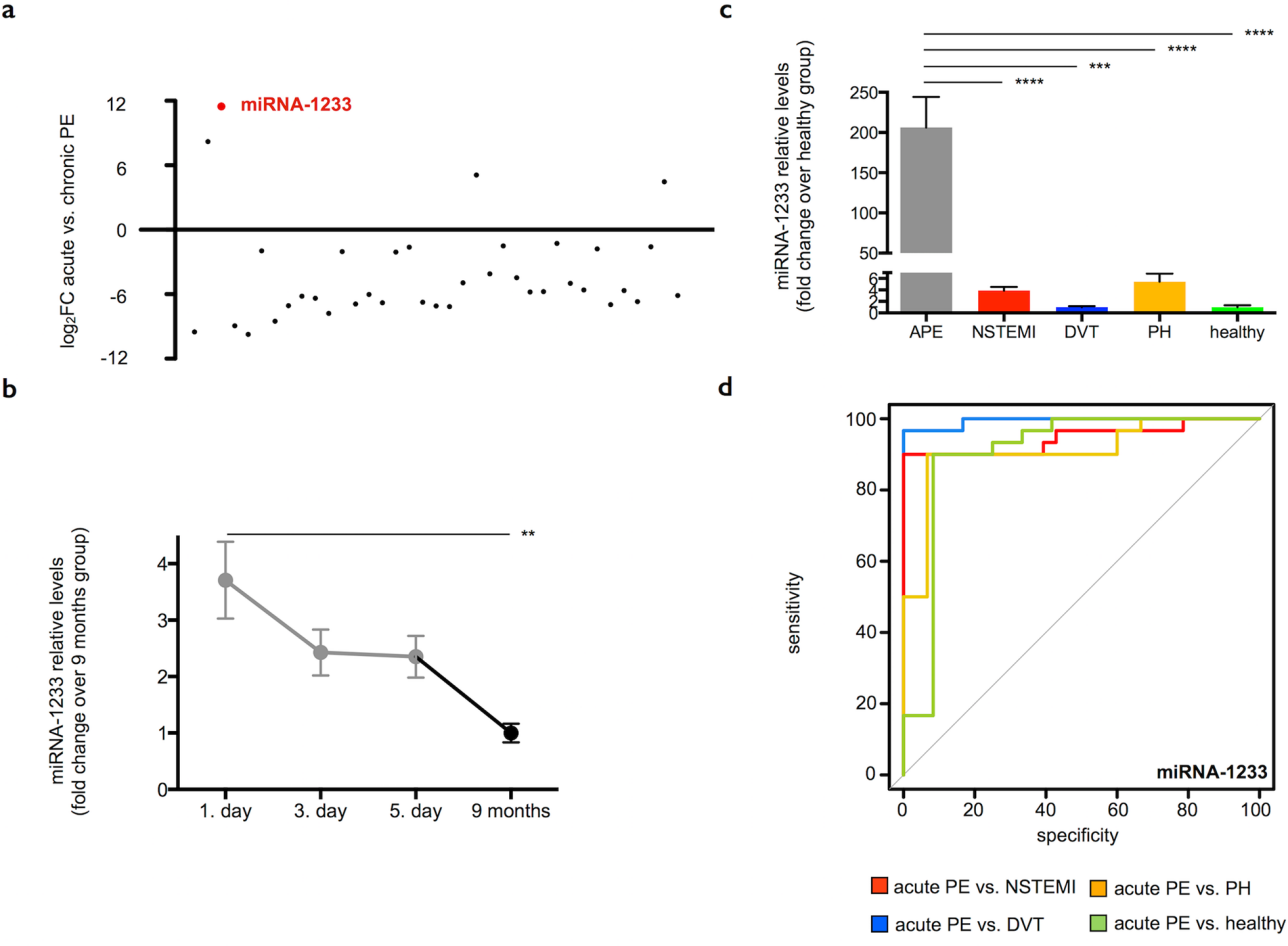
miRNA-1233. **a** microRNA (miRNA) array screening. We randomly selected 16 out of 30 pulmonary embolism (PE) patients and screened their sera from the acute (1st day) and chronic stage (9 months) for 754 miRNAs. The 37 most differentially expressed miRNAs (nominal P value ≤ 0.05) were displayed. miRNA-1233 (*red dot*) displayed the highest fold change on the 1st day (log_2_FC 11.5 on 1st day vs. 9 months, p < 0.004) and was consequently subjected to real-time quantitative polymerase chain reaction (RT-qPCR) validation. **b** miRNA RT-qPCR validation. Serum miRNA-1233 levels during the time course of acute PE (1st day: presentation at the emergency department, 3rd and 5th day during the hospital stay (n = 30) and 9 ± 1.5 months thereafter (n = 22). Values represent relative levels with the “9 months” group set as 1 (mean ± SEM, **p < 0.01). **c** Serum miRNA-1233 levels from acute pulmonary embolism patients (APE, 1st day, n = 30) in comparison to patients with acute non ST-segment elevation myocardial infarction (NSTEMI, n = 30), acute deep vein thrombosis without concomitant PE (DVT, n = 6), chronic non-thromboembolic pulmonary hypertension (PH, n = 15) and healthy individuals (n = 12). Values represent relative levels with the “healthy” group set as 1 (mean ± SEM, ***p < 0.001, ****p < 0.0001). **d** Receiver operating characteristic (ROC) curve analysis for miRNA-1233 to discriminate acute PE (1st day) from acute NSTEMI (*red line*, area under the curve (AUC) 0.95, p < 0.001, sensitivity 90 % and specificity 100 %), acute DVT (*blue line*, AUC 0.99, p < 0.001, sensitivity 97 % and specificity 100 %), chronic PH (*orange line*, AUC 0.91, p < 0.001, sensitivity 90 % and specificity 93 %) and healthy individuals (*green line*, AUC 0.91, p < 0.001, sensitivity 90 % and specificity 92 %)

We first randomly selected 16 out of 30 PE patients and screened their sera from the acute phase (1st day) and from the chronic stage (9 months) for 754 miRNAs using TaqMan human miRNA arrays. We found 37 miRNAs to be differentially regulated with a nominally significant *P* value (<0.05, 1st day vs. 9 months, Fig. 2a). Across all screened miRNAs, miRNA-1233 displayed the highest fold change at the acute (1st day) in comparison to the chronic stage (9 months) (log_2_FC 11.5, *P* < 0.004). We then validated our array findings on all enrolled PE patients from all time points by TaqMan-based miRNA real-time quantitative polymerase chain reaction (RT-qPCR). We confirmed that miRNA-1233 displayed highest serum levels on the first day, its levels then decreased on the third and fifth day and were lowest at the chronic stage of the disease (FC 3.7 on 1st day vs. 9 months, p < 0.01, Fig. 2b). Our findings show that miRNA-1233 was up-regulated in the serum of acute PE patients, indicating that it could represent a marker for diagnosing acute PE.

### Sensitivity and specificity of miRNA-1233 for diagnosing acute PE

We then tested miRNA-1233 specificity and sensitivity for diagnosing acute PE and compared miRNA-1233 serum levels from acute PE patients to levels from patients with acute NSTEMI, acute DVT, chronic non-thromboembolic PH and healthy individuals by TaqMan-based miRNA RT-qPCR. Patients’ characteristics are shown in Table 2. We found significantly higher levels of miRNA-1233 in the serum of acute PE patients in comparison to all other groups (PE 1st day FC 206.3, NSTEMI FC 3.9, DVT FC 1, PH FC 5.4 vs. healthy, Fig. 2c). Receiver operating characteristic (ROC) analysis revealed that miRNA-1233 differentiated acute PE (1st day) from acute NSTEMI, DVT, PH patients and healthy controls with high sensitivity (90, 97, 90, and 90 %, respectively) and specificity (100, 100, 93, and 92 %, respectively, Fig. 2c). Detailed ROC statistics are shown in Table 3. Taken together, miRNA-1233 distinguished acute PE (1st day) from acute NSTEMI, its most frequent differential diagnosis, with high specificity and sensitivity.

**Table 3:** Receiver operating characteristic curves

	AUC	95 % CI	p	Cut-off	Sensitivity (%)	95 % CI	Specificity (%)	95 % CI	LR+	LR−
*Acute PE vs. healthy*										
miR-27a	0.79	0.65–0.93	<0.01	0.63	67	50–83	92	75–100	8	0.4
miR-134	0.84	0.71–0.96	<0.001	0.51	83	70–97	83	58–100	5	0.2
miR-1233	0.91	0.82–0.99	<0.001	0.53	90	77–100	92	75–100	10.8	0.1
miR-27a + miR-134	0.88	0.78–0.98	<0.001	0.47	90	77–100	83	58–100	5.4	0.1
miR-27a + miR-1233	0.89	0.79–0.99	<0.001	0.48	97	90–100	83	58–100	5.8	0
miR-134 + miR-1233	0.88	0.77–0.98	<0.001	0.40	93	83–100	75	50–100	3.7	0
miR-27a + miR-134 + miR-1233	0.90	0.81–0.99	<0.001	0.50	90	80–100	83	58–100	5.4	0.1
*Acute PE vs. NSTEMI*										
miR-27a	0.78	0.66–0.90	<0.001	0.46	63	43–80	89	79–100	5.9	0.4
miR-134	0.78	0.66–0.90	<0.001	0.74	83	70–97	64	46–82	2.3	0.3
miR-1233	0.95	0.89–1.00	<0.001	0.55	90	80–100	100	100–100	∞	0.1
miR-27a + miR-134	0.80	0.68–0.91	<0.001	0.34	87	73–97	61	43–79	2.2	0.2
miR-27a + miR-1233	0.96	0.91–1.00	<0.001	0.69	90	77–100	100	100–100	∞	0.1
miR-134 + miR-1233	0.95	0.89–1.00	<0.001	0.54	90	77–100	100	100–100	∞	0.1
miR-27a + miR-134 + miR-1233	0.97	0.93–1.00	<0.001	0.71	90	77–100	100	100–100	∞	0.1
*Acute PE vs. DVT*										
miR-27a	0.83	0.67–0.98	<0.05	0.79	73	57–90	100	100–100	∞	0.3
miR-134	0.84	0.72–0.99	<0.01	0.74	80	67–93	100	100–100	∞	0.2
miR-1233	0.99	0.97–1.0	<0.001	0.72	97	90–100	100	100–100	∞	0
miR-27a + miR-134	0.92	0.83–1.0	<0.001	0.63	87	73–97	100	100–100	∞	0.1
miR-27a + miR-1233	0.99	0.97–1.0	<0.001	0.72	97	90–100	100	100–100	∞	0
miR-134 + miR-1233	0.99	0.97–1.0	<0.001	0.72	97	90–100	100	90–100	∞	0
miR-27a + miR-134 + miR-1233	0.99	0.97–1.0	<0.01	0.72	97	90–100	100	100–100	∞	0
*Acute PE vs. PHT*										
miR-27a	0.66	0.50–0.82	0.085	0.66	67	50–83	80	60–100	3.3	0.4
miR-134	0.67	0.51–0.83	0.062	0.61	70	53–87	73	47–93	2.6	0.4
miR-1233	0.91	0.83–0.99	<0.001	0.43	90	80–100	93	80–100	13.5	0.1
miR-27a + miR-134	0.66	0.50–0.82	0.085	0.59	70	53–87	73	47–93	2.6	0.4
miR-27a + miR-1233	0.92	0.84–1.0	<0.001	0.42	90	77–100	93	80–100	13.5	0.1
miR-134 + miR-1233	0.91	0.83–0.99	<0.001	0.42	90	77–100	93	80–100	13.5	0.1
miR-27a + miR-134 + miR-1233	0.90	0.81–0.99	<0.001	0.46	90	80–100	93	80–100	13.5	0.1

Receiver operating characteristic (ROC) curves. Cut-off is defined as the Youden’s index which represents the point on the curve furthest away from the 45 degree line

*AUC* area under the curve, *CI* confidence interval, *LR* + positive likelihood ratio, *LR*- negative likelihood ratio, *miR* microRNA, *PE* pulmonary embolism, *NSTEMI* acute non-ST segment myocardial infarction, *DVT* acute deep vein thrombosis without concomitant PE, *PH* chronic non-thromboembolic pulmonary hypertension

### miRNA profile/signature

**Fig. 3:**
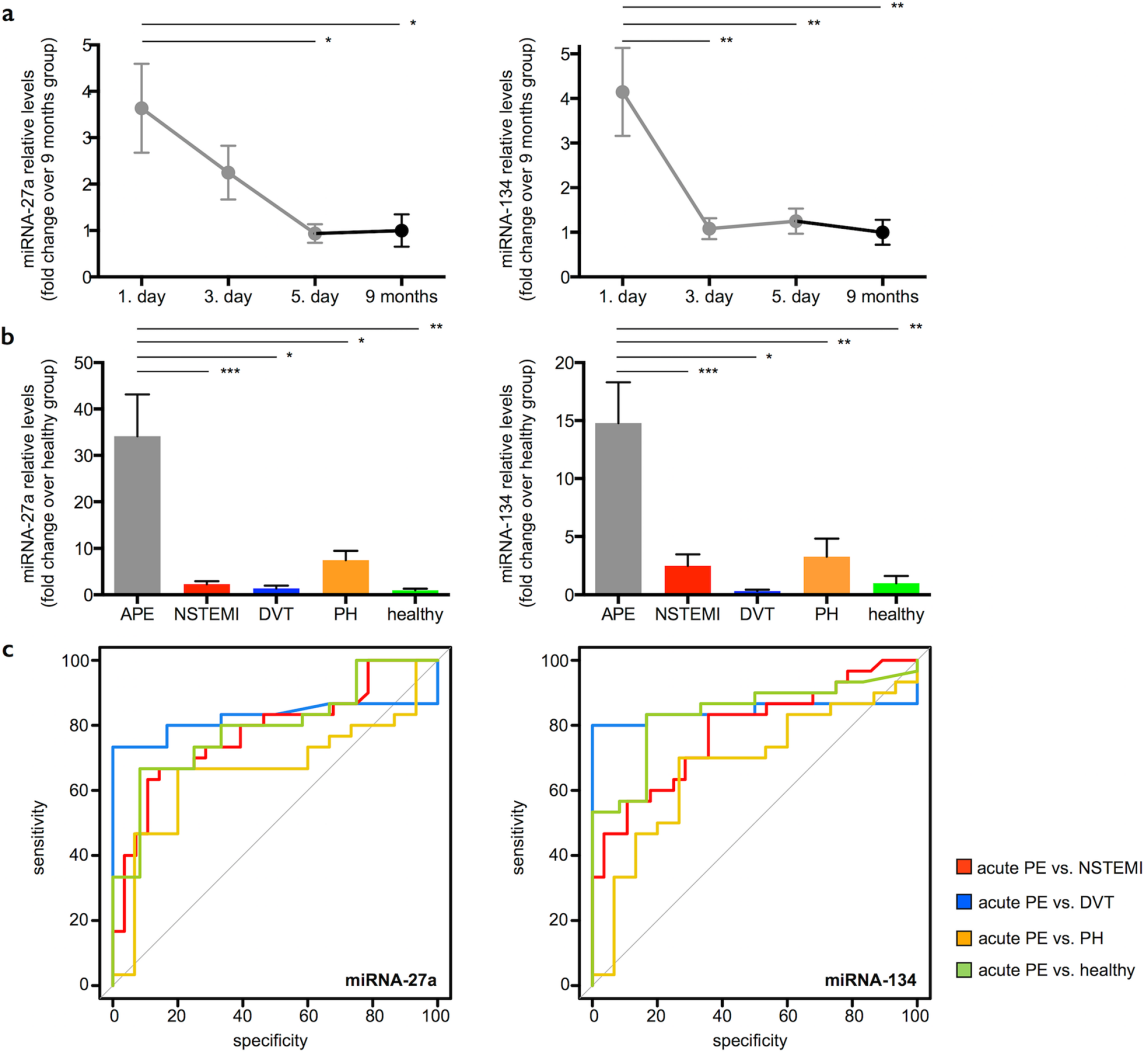
miRNA-27a and 134. **a** Serum miRNA-27a and 134 levels during the time course of acute pulmonary embolism (APE, 1st day: presentation at the emergency department, 3rd and 5th day during the hospital stay (n = 30) and 9 ± 1.5 months thereafter (n = 22). Values represent relative levels with the pulmonary embolism (PE) “9 months” group set as 1 (mean ± SEM, *p < 0.05, **p < 0.01). **b** Serum miRNA-27a and 134 levels from acute PE patients (APE, 1st day, n = 30) in comparison to patients with acute non ST-segment elevation myocardial infarction (NSTEMI, n = 30), acute deep vein thrombosis without concomitant PE (DVT, n = 6), chronic non-thromboembolic pulmonary hypertension (PH, n = 15) and healthy individuals (n = 12). Values represent relative levels with the “healthy” group set as 1 (mean ± SEM, *p < 0.05, **p < 0.01, ***p < 0.001). **c** ROC (Receiver operating characteristic) curve analysis for miRNA-27a and 134 to discriminate acute PE (1st day) from acute NSTEMI (*red line*, area under the curve (AUC) 0.78, 0.78, p < 0.001, < 0.001, respectively), acute DVT (*blue line*, AUC 0.83, 0.84, p < 0.05, < 0.01, respectively), chronic PH (*orange line*, AUC 0.66, 0.67, p = 0.09, 0.06, respectively) and healthy individuals (*green line*, AUC 0.79, 0.84, p < 0.01, < 0.001, respectively)

**Fig. 4:**
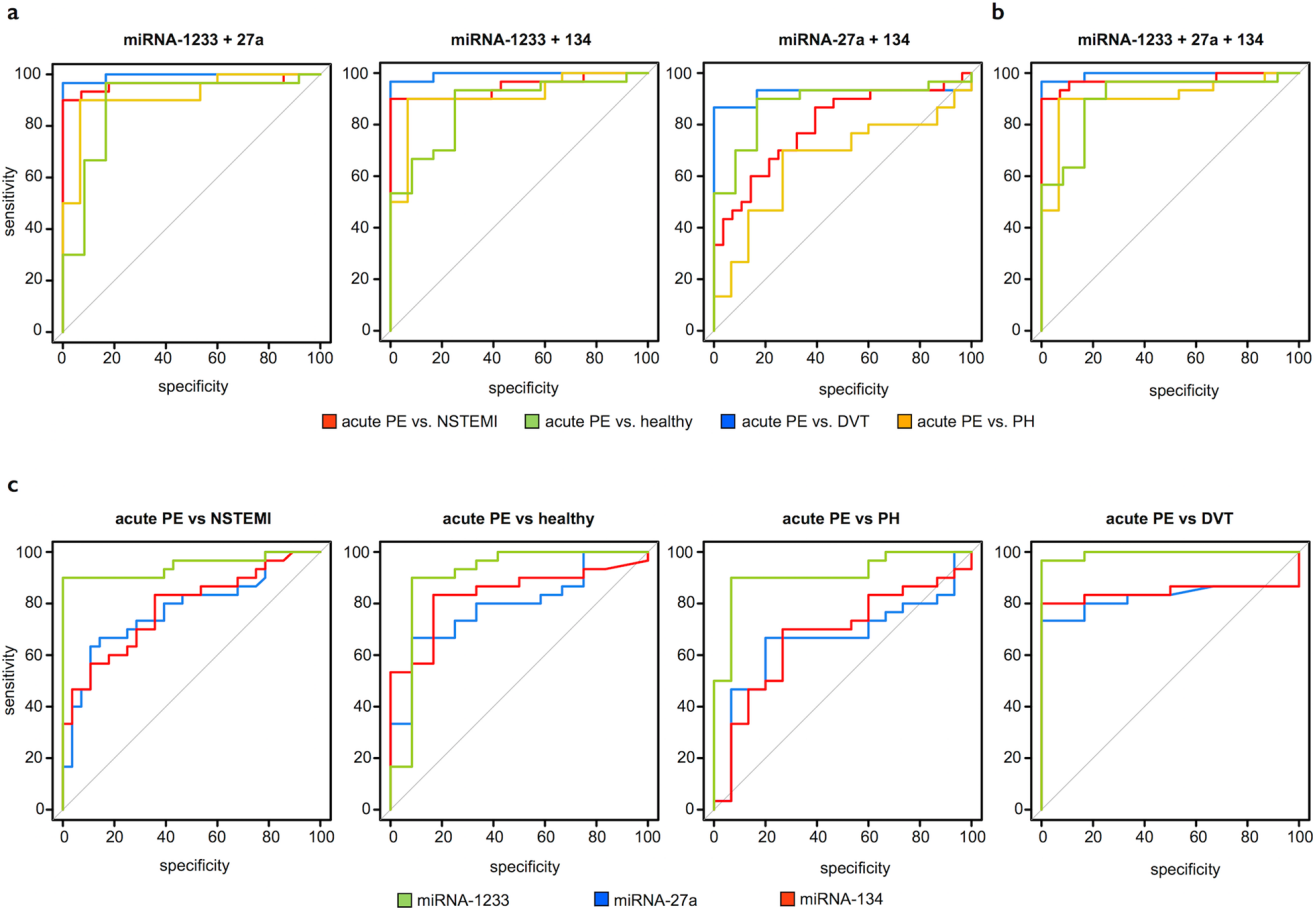
Receiver operating characteristic curve analysis. ROC (Receiver operating characteristic) curve analysis for **a** combining two microRNAs (miRNA) or **b** all three miRNAs to discriminate acute pulmonary embolism (PE, 1st day) from acute non-ST segment myocardial infarction (NSTEMI, *red line*), acute deep vein thrombosis without concomitant PE (DVT, *blue line*), chronic non-thromboembolic pulmonary hypertension (PH, *orange line*) and healthy individuals (*green line*). **c** ROC curve analysis for comparing miRNAs (*green line*: miRNA-1233, *red line*: miRNA-134, *blue line*: miRNA-27a) to discriminate acute PE (1st day) from acute NSTEMI, acute DVT without concomitant PE, chronic non-thromboembolic PH and healthy individuals

We further identified two additional miRNAs, miRNA-27a and miRNA-134, which exhibited highest serum levels in PE patients on the first day and lowest levels at 9 months (FC 3.6, 4.1 on 1st day vs. 9 months, p < 0.05, p < 0.01, respectively, Fig. 3a). Similar to miRNA-1233, we found significantly higher levels of miRNAs -27a and 134 in the serum of acute PE patients in comparison to all other groups (PE 1st day FC 34.1, 14.8; NSTEMI FC 2.3, 2.5; DVT FC 1.4, 0.3; PH FC 7.5, 3.3 vs. healthy, respectively, Fig. 3b). ROC analysis revealed that miRNA-27a differentiated acute PE (1st day) from NSTEMI, DVT, PH patients and healthy controls with 63, 73, 67, and 67 % sensitivity, and 89, 100, 80, and 92 % specificity, respectively (Fig. 3c). Accordingly, miRNA-134 differentiated acute PE (1st day) from NSTEMI, DVT, PH patients and healthy controls with 83, 80, 70, and 83 % sensitivity, and 64, 100, 73, and 83 % specificity, respectively (Fig. 3c). Detailed ROC statistics are shown in Table 3. We then tested, whether or not a combination of miRNAs (serum miRNA profile/signature) improved diagnostic sensitivity and specificity. Neither combining miRNA-1233 with either miRNA-27a or miRNA-134 (Fig. 4a; Table 3), nor combining all three miRNAs improved sensitivity and specificity for accurately differentiating acute PE from healthy individuals and acute NSTEMI patients (Fig. 4b; Table 3). We then directly compared all three miRNA and found that miRNA-1233 was always superior to miRNAs-27a and miRNA-134 for accurately distinguishing acute PE from acute NSTEMI, acute DVT and PH patients and from healthy individuals (Fig. 4c; Table 3). In summary, miRNA-1233 alone yielded a superior capability of distinguishing acute PE from healthy individuals and the described diseases in comparison to miRNA-27a and miRNA-134 (Table 3). A signature, comprising miRNAs-1233, 27a and 134 could not further increase sensitivity and specificity, and was consequently not superior to miRNA-1233 alone.

## Discussion

A timely diagnosis of PE remains to be a challenging clinical problem [3, 32]. In fact, recent studies have revealed that up to one third of all PE cases are missed at the emergency department [33]. Here, we screened sera of PE patients for more than 750 miRNA by using miRNA arrays and found significantly elevated levels of miRNA-1233 in patients with acute PE (vs. chronic PE). We validated these array findings by individual TaqMan qPCR and found—as compared to acute PE patients—markedly lower miRNA-1233 levels in patients with NSTEMI, DVT, PH, as well as in healthy individuals. Importantly, miRNA-1233 distinguished acute PE from acute NSTEMI, its most frequent differential diagnosis, with high specificity and sensitivity.

Pulmonary embolism is a frequent emergency affecting 100–300/100,000 individuals per year in industrialized nations [3]. Like acute MI patients, patients with acute PE present symptoms like chest pain and shortness of breath. Consequently, acute PE and acute MI are among the most frequent differential diagnosis for shortness of breath and chest pain [3, 4], and distinguishing these diseases can be difficult due to a similar clinical presentation. However, distinguishing PE from MI early on is crucial because these different pathologies require distinct treatments, which, when applied in a timely fashion, significantly enhance patient survival [34–37]. PE treatment is mainly conservative and focuses on anticoagulation, while in MI an invasive re-vascularization therapy is often pursued [38].

To date, there are no specific biomarkers for an early accurate detection of acute PE. High sensitivity troponins and D-dimers are often used for diagnosing MI or PE, respectively. However, these markers do not perfectly discriminate the two conditions. Specifically, elevated D-dimer levels can also be found in MI patients [39], as well as in a number of other conditions [1]. Vice versa, elevated troponin levels can also be detected in acute PE, especially if the right heart is affected (acute cor pulmonale) [40].

Rapidly and accurately diagnosing acute PE would be an extremely helpful tool, especially at emergency departments. Ideally, miRNA-1233 could identify acute PE patients early as a bedside test so that correct treatment could be initiated timely, consequently reducing mortality and morbidity. As of now, most laboratories use RT-qPCR-based methods for detecting serum miRNAs. RT-qPCR is very sensitive on one hand, but also difficult to standardize on the other. To date, a house keeping miRNA to normalize miRNA content to is lacking. The current practice of supplementing external controls (for instance spiking of miRNAs for normalization in PCR-based measurements) might not be sufficient enough to provide an accurate bedside measurement of circulating miRNAs. For the clinical routine, well-defined cutoff values and reliable measurements are most crucial and would be needed.

To date, there is one other study that also evaluated serum miRNAs for diagnosing PE [18]. We reproduced the finding of Xiao et al. that miRNA-134 is up-regulated in acute PE patients making miRNA-134 an even more promising marker, since our findings are the first replication on an independent cohort. However, the study did not report on miRNA-1233 or on miRNA-27a, and nor did it show miRNA-134 specificity to distinguish PE from other relevant differential diagnosis like NSTEMI.

Interpretations of our study results are limited mainly due to (1) a rather small sample size (n = 30 in each the PE and NSTEMI group) and (2) a highly selected, very morbid group of PE patients with a high thrombus burden. Our findings need to be replicated on a larger cohort of patients and also on less morbid PE patients with peripheral rather than central emboli.

## Conclusions

Circulating miRNA-1233 allows distinguishing acute PE from acute NSTEMI and healthy individuals with high specificity and sensitivity, and consequently appears to be a promising marker for accurately diagnosing acute PE.
